# Artificial intelligence literacy and associated factors among gastroenterologists in China: findings from a multicenter survey with nationwide geographic coverage

**DOI:** 10.3389/fpubh.2026.1967305

**Published:** 2026-10-21

**Authors:** Yangyang Zhang, Wenhao Zhang, Canlin Wang, Hewei Ma, Lanshuo Hu, Yiqing Mao, Jian Lan, Xudong Tang, Yingpan Zhao

**Affiliations:** 1 Institute of Digestive Diseases, Xiyuan Hospital, China Academy of Chinese Medical Sciences, Beijing, China; 2 Guang’anmen Hospital, China Academy of Chinese Medical Sciences, Beijing, China; 3 Department of Gastroenterology, Tongde Hospital of Zhejiang Province, Hangzhou, China; 4 Taizhou Hospital of The First Affiliated Hospital of Zhejiang University School of Medicine, Taizhou, China

**Keywords:** AI literacy, artificial intelligence, China, cross-sectional study, gastroenterologists

## Abstract

**Background:**

Artificial intelligence (AI) is increasingly integrated into healthcare, making AI literacy an emerging core competency for physicians. However, AI literacy among Chinese gastroenterologists has not been systematically investigated. This survey assessed AI literacy among Chinese gastroenterologists and examined its associated factors.

**Methods:**

We conducted a multicenter cross-sectional survey with nationwide geographic coverage among gastroenterologists in China. The study was implemented from February to June 2026 following ethical approval. Participants were recruited from 96 hospitals across 30 provincial-level regions and completed a structured online questionnaire assessing AI literacy using the Medical Artificial Intelligence Readiness Scale for Medical Students (MAIRS-MS). Following univariable analyses, multiple linear regression was used to identify factors independently associated with AI literacy.

**Results:**

A total of 1,125 valid questionnaires were analyzed. The median overall AI literacy score was 82 (IQR, 69–96). Median scores for the Cognition, Ability, Vision, and Ethics domains were 28, 31, 11, and 12, respectively; after normalization to a 0–100 scale, the corresponding scores were 62.5%, 71.9%, 66.7%, and 75.0%. In the adjusted analysis, more frequent AI tool use was associated with progressively higher AI literacy scores compared with rare or no use (2–3 times/week: B = 3.47; 4–5 times/week: B = 5.86; almost daily: B = 8.00; all *p* ≤ 0.020). AI-related training (B = 7.61, *p* < 0.001) and self-reported AI use in medical practice (B = 5.93, *p* < 0.001) were also associated with higher scores. Participants from central China had higher scores (*p* < 0.001), whereas those from western China had lower scores (*p* = 0.025), compared with those from eastern China.

**Conclusion:**

Chinese gastroenterologists reported varying levels of perceived AI readiness across MAIRS-MS domains, with the lowest normalized scores observed in Cognition and Vision. Self-reported AI literacy was associated with educational attainment, geographic region, AI tool use frequency, AI-related training, and AI use in medical practice. These findings highlight the potential relevance of structured and regularly updated AI education, while longitudinal studies incorporating objective competency assessments are needed to clarify these associations and determine whether educational interventions improve physicians’ AI-related knowledge and skills.

## 1 Introduction

The rapid development of generative artificial intelligence (AI), particularly large language models, is reshaping clinical practice, medical education, and healthcare delivery (1). Its growing use in healthcare has also raised concerns regarding algorithmic bias, data privacy, and ethical accountability (2). For physicians, the central question is therefore shifting from whether AI should be adopted to how it can be understood, critically evaluated, and appropriately integrated into clinical practice. Accordingly, AI literacy has attracted increasing attention in medical education and public health and is emerging as an important professional competency (3).

AI literacy generally refers to the capacity to understand and interact with AI systems and critically evaluate the information or outputs they generate. It encompasses four principal domains: cognition, ability, vision, and ethics (4). Previous studies have extensively examined physicians’ awareness of, attitudes towards, and willingness to adopt AI, generally reporting favorable views. However, willingness to accept or use AI does not necessarily indicate adequate AI literacy (5). AI acceptance and intention to use primarily reflect attitudes and behavioral willingness. By contrast, AI literacy encompasses the knowledge, critical judgment, practical skills, and ethical awareness required to work effectively in AI-supported healthcare environments. It is therefore increasingly recognized as a foundation for the responsible use of AI and for integrating AI education into professional training.

Several standardized instruments have been developed in response to the growing need to assess AI literacy (6). Among them, the Medical Artificial Intelligence Readiness Scale for Medical Students (MAIRS-MS) is a widely used and rigorously validated instrument in medical education (7). A recent systematic review of 21 studies using the MAIRS-MS found that the available evidence was concentrated in countries such as Türkiye, Iran, and Malaysia. By comparison, evidence from China, the United States, and Europe remained limited (8). This uneven geographic distribution restricts the generalizability of existing findings across countries, healthcare systems, and clinical contexts.

This evidence gap is particularly relevant to gastroenterology, a specialty in which the clinical application of AI has advanced rapidly. AI has been applied to gastrointestinal lesion detection during endoscopy, cancer screening, computer-assisted pathological diagnosis, and disease risk prediction. In recent years, several AI-assisted systems have also been incorporated into routine clinical workflows (9). Nevertheless, AI literacy among gastroenterologists remains insufficiently characterized, and no study has systematically evaluated it among gastroenterologists in China.

We therefore conducted a multicenter cross-sectional survey to assess AI literacy among Chinese gastroenterologists and identify factors associated with it. This study provides evidence on the current level and distribution of AI literacy within this professional group. The findings may inform the development of targeted AI education and training programs and support competency-based workforce development in gastroenterology.

## 2 Methods

### 2.1 Study design and setting

This multicenter cross-sectional study was reported in accordance with the Strengthening the Reporting of Observational Studies in Epidemiology (STROBE) guidelines (10). This multicenter cross-sectional survey was conducted as part of a project implemented from June 2025 to June 2026. During the preparatory phase in 2025, the questionnaire was developed and refined, and the study underwent the required ethical review procedures. Following ethical approval in 2026, eligible gastroenterologists were recruited and invited to complete the questionnaire, and participant data were collected from February to June 2026. The study protocol was approved by the Ethics Committee of Xiyuan Hospital, China Academy of Chinese Medical Sciences (approval no. 2026XLA092-1). All participants provided electronic informed consent before participating in the survey.

### 2.2 Participants

Participants were eligible if they met all of the following criteria: (1) were aged 18–70 years; (2) were currently involved in the clinical diagnosis and management of gastrointestinal diseases; (3) held a valid Chinese physician qualification certificate; (4) were able to access and complete the online questionnaire using a smartphone or computer; (5) had sufficient reading comprehension to understand and complete the questionnaire; and (6) provided electronic informed consent.

Participants were excluded if they (1) were unable to complete the questionnaire independently because of a psychiatric disorder, cognitive impairment, or physical disability; (2) withdrew before completing the survey; or (3) submitted a duplicate response.

### 2.3 Data collection

The questionnaire comprised two sections. The first section collected demographic, professional, and AI-related characteristics, including sex, age, educational attainment, professional title, province of practice, years in practice, frequency of AI tool use, previous AI-related training, and self-reported use of AI in medical practice. These AI-related exposure items were based on participants’ self-report and did not distinguish among specific types of AI tools, training formats, or clinical applications. The exact wording and response options are provided in the Supplementary Questionnaire. The second contained the validated Chinese version of MAIRS-MS, which was used with permission from its developers (11). The scale has a possible total score ranging from 22 to 110 and comprises four domains: Cognition (8–40 points), Ability (8–40 points), Vision (3–15 points), and Ethics (3–15 points). Higher scores indicate greater self-reported AI literacy.

The study was supported by a national academic society. With the assistance of this society, physicians from 96 hospitals were invited to serve as local coordinators, and hospital-based recruitment teams were established. The coordinators distributed the questionnaire to gastroenterologists at their respective hospitals who met the eligibility criteria. Each hospital was asked to distribute 15 questionnaires. All participating institutions were tertiary hospitals located across 30 provincial-level regions of China, excluding Tibet, Hong Kong, Macao, and Taiwan (Supplementary Table S1). Based on their levels of economic development, these regions were categorized as eastern, central, western, or northeastern China.

To ensure data quality, the survey platform restricted each participant to a single submission based on their IP address, mobile phone number, and device identifier. A quality-control item was embedded in the questionnaire, and responses that failed this item were excluded. Participants who selected the lowest response option for every scale item were classified as exhibiting invariant response patterns and were also excluded. In addition, questionnaires completed in less than 1 min were excluded from the analysis.

### 2.4 Sample size estimation

The required sample size was estimated using G*Power version 3.1. An *a priori* power analysis for multiple linear regression was performed using an F-test. With a two-sided significance level of 0.05, a statistical power of 90%, and an assumed effect size of *f*^2^ = 0.05, the minimum required sample size was estimated to be 416 participants. After allowing for an anticipated invalid-response rate of 20%, the target sample size was increased to at least 520 participants.

### 2.5 Statistical analysis

Data were managed using WPS Office and analyzed using R version 4.2.2. Continuous variables were summarized as means with standard deviations or medians with interquartile ranges (IQRs), as appropriate, and categorical variables as frequencies and percentages. Between-group differences were assessed using the Mann–Whitney U test, Kruskal-Wallis test, chi-squared test, or Fisher’s exact test, as appropriate. Significant Kruskal-Wallis tests were followed by Dunn’s pairwise comparisons with Holm adjustment. To facilitate comparisons across MAIRS-MS domains with different score ranges, scores were normalized to a 0-100 scale using [(observed score − minimum possible score)/(maximum possible score − minimum possible score)] × 100.

Univariable analyses of AI literacy were considered exploratory. As a sensitivity analysis for multiple testing, the 45 corresponding *p* values were adjusted using the Benjamini-Hochberg false discovery rate (FDR) procedure. Effect sizes were estimated using rank-biserial correlations for two-group comparisons and Kruskal–Wallis eta-squared (*η*^2^H) for multicategory comparisons; 95% confidence intervals (CIs) for *η*^2^H were obtained using percentile bootstrap resampling with 5,000 replications.

Multiple linear regression was used to examine factors associated with AI literacy. The model included sex, age group, highest educational attainment, professional title, geographic region, frequency of AI tool use, AI-related training, and clinical AI use, selected *a priori* irrespective of univariable statistical significance. AI tool use frequency was modeled categorically, with “rarely or never” as the reference category. Model assumptions and diagnostics were assessed, and HC3 heteroskedasticity-robust standard errors were used because heteroskedasticity was detected. Unstandardized coefficients (B), robust 95% CIs, and *p* values were reported. An ordinal specification of AI tool use frequency was additionally examined as a sensitivity analysis. Because individual hospital identifiers were not collected, hospital-level clustering could not be incorporated into the analysis. No data were missing for variables reported in Tables 1–3 or included in the regression model; therefore, no missing-data imputation was required.

**Table 1:** Characteristics of the study participants.

Variables	Total (*n* = 1,125)
Sex, *n* (%)	
Male	440 (39.11%)
Female	685 (60.89%)
Age, *n* (%)	
≤40 years	672 (59.73%)
>40 years	453 (40.27%)
Highest degree, *n* (%)	
Bachelor’s degree	302 (26.84%)
Master’s degree	598 (53.16%)
Doctoral degree	225 (20.00%)
Professional title, *n* (%)	
Chief Physician	204 (18.13%)
Associate Chief Physician	301 (26.76%)
Attending Physician	357 (31.73%)
Physician	263 (23.38%)
Region, *n* (%)	
Central Region	379 (33.69%)
Western Region	267 (23.73%)
Eastern Region	420 (37.33%)
Northeastern Region	59 (5.24%)
Years in practice, *n* (%)	
<5 years	235 (20.89%)
5–10 years	222 (19.73%)
>10 years	668 (59.38%)
Frequency of AI tool use, *n* (%)	
Rarely or never	184 (16.36%)
2–3 times per week	503 (44.71%)
4–5 times per week	143 (12.71%)
Almost every day	295 (26.22%)
Received AI-related training, *n* (%)	
Yes	427 (37.96%)
No	698 (62.04%)
Use of AI tools in clinical practice, *n* (%)	
Yes	716 (63.64%)
No	409 (36.36%)

*n*: number; AI: artificial intelligence.

**Table 2:** AI literacy and associated factors among Chinese gastroenterologists.

Variables	Cognition		Ability		Vision		Ethics		Overall	
	Median (IQR)	*p*-value	Median (IQR)	*p*-value	Median (IQR)	*p*-value	Median (IQR)	*p*-value	Median (IQR)	*p*-value
Sex, *n* (%)										
Male	28.00 (24.00, 34.00)	0.832	31.00 (25.00, 36.00)	0.647	11.00 (9.00, 13.00)	0.704	12.00 (10.00, 15.00)	**0.034**	81.00 (69.00, 95.00)	0.720
Female	28.00 (24.00, 35.00)		31.00 (26.00, 36.50)		11.00 (9.00, 13.00)		12.00 (11.00, 15.00)		83.00 (70.00, 97.00)	
Age, *n* (%)										
≤40 years	28.00 (24.00, 34.00)	0.613	32.00 (26.00, 36.00)	0.192	11.00 (9.00, 13.00)	0.199	12.00 (10.00, 15.00)	0.985	83.00 (70.00, 96.00)	0.325
>40 years	28.00 (24.00, 34.00)		30.00 (25.00, 36.00)		10.00 (9.00, 13.00)		12.00 (10.00, 15.00)		81.00 (69.00, 96.50)	
Highest degree, *n* (%)										
Bachelor’s degree	32.00 (24.75, 40.00)	**<0.001**	32.00 (27.00, 40.00)	**<0.001**	12.00 (9.00, 15.00)	**<0.001**	14.50 (11.00, 15.00)	**<0.001**	89.00 (72.75, 109.25)	**<0.001**
Master’s degree	26.00 (24.00, 32.00)		30.00 (25.00, 33.00)		10.00 (9.00, 12.00)		12.00 (10.00, 15.00)		79.00 (68.00, 89.00)	
Doctoral degree	28.00 (24.00, 33.50)		32.00 (26.00, 35.50)		11.00 (9.00, 12.00)		12.00 (10.00, 15.00)		83.00 (71.00, 94.50)	
Professional title, *n* (%)										
Chief Physician	27.00 (24.00, 34.00)	**<0.001**	30.00 (26.00, 36.00)	**<0.001**	11.00 (9.00, 13.00)	**<0.001**	12.00 (10.00, 15.00)	**<0.001**	80.00 (69.25, 95.00)	**<0.001**
Associate Chief Physician	28.00 (24.00, 32.00)		30.00 (24.00, 35.00)		10.00 (9.00, 12.00)		12.00 (10.00, 15.00)		81.00 (68.00, 90.50)	
Attending Physician	27.00 (24.00, 32.00)		30.00 (25.00, 34.00)		10.00 (9.00, 12.00)		12.00 (9.00, 15.00)		80.00 (68.00, 91.00)	
Physician	31.00 (25.00, 40.00)		32.00 (28.00, 40.00)		12.00 (9.00, 15.00)		14.00 (12.00, 15.00)		88.00 (75.00, 108.00)	
Region, *n* (%)										
Central Region	32.00 (26.00, 40.00)	**<0.001**	33.00 (28.00, 40.00)	**<0.001**	12.00 (9.00, 15.00)	**<0.001**	15.00 (12.00, 15.00)	**<0.001**	89.00 (75.00, 110.00)	**<0.001**
Western Region	25.00 (23.00, 32.00)		29.00 (24.00, 32.00)		10.00 (9.00, 12.00)		12.00 (9.00, 15.00)		76.00 (66.00, 88.00)	
Eastern Region	27.00 (24.00, 32.00)		31.00 (26.00, 33.00)		10.00 (9.00, 12.00)		12.00 (11.00, 15.00)		81.00 (69.00, 90.00)	
Northeastern Region	27.00 (23.00, 31.00)		30.00 (24.00, 32.00)		10.00 (9.00, 12.00)		12.00 (10.00, 15.00)		79.00 (68.00, 88.00)	
Years in practice, *n* (%)										
<5 years	28.00 (24.00, 33.00)	0.746	32.00 (27.00, 35.00)	0.553	11.00 (9.00, 13.00)	0.546	12.00 (11.00, 15.00)	0.952	83.00 (72.00, 92.00)	0.580
5–10 years	27.00 (24.00, 34.25)		31.50 (25.00, 38.25)		11.00 (9.00, 13.00)		12.00 (9.00, 15.00)		81.00 (69.00, 97.25)	
>10 years	28.00 (24.00, 34.00)		30.50 (25.00, 36.00)		11.00 (9.00, 13.00)		12.00 (10.00, 15.00)		82.00 (69.00, 97.00)	
Frequency of AI tool use, *n* (%)										
Rarely or never	25.00 (21.25, 32.00)	**<0.001**	26.50 (24.00, 32.00)	**<0.001**	9.00 (9.00, 12.00)	**<0.001**	12.00 (9.00, 15.00)	**<0.001**	73.00 (65.00, 88.00)	**<0.001**
2–3 times per week	27.00 (24.00, 32.00)		30.00 (25.00, 34.00)		10.00 (9.00, 12.00)		12.00 (10.00, 15.00)		80.00 (69.00, 91.00)	
4–5 times per week	29.00 (25.00, 35.00)		32.00 (27.00, 37.00)		11.00 (9.00, 12.00)		13.00 (12.00, 15.00)		83.00 (77.00, 99.00)	
Almost every day	32.00 (25.00, 40.00)		33.00 (28.00, 40.00)		12.00 (9.00, 15.00)		14.00 (12.00, 15.00)		88.00 (76.00, 108.00)	
Received AI-related training, *n* (%)										
Yes	32.00 (26.00, 40.00)	**<0.001**	33.00 (29.00, 40.00)	**<0.001**	12.00 (10.00, 15.00)	**<0.001**	14.00 (12.00, 15.00)	**<0.001**	89.00 (77.00, 108.00)	**<0.001**
No	26.00 (23.00, 31.00)		29.00 (24.00, 32.00)		10.00 (9.00, 12.00)		12.00 (9.00, 15.00)		77.50 (66.00, 88.00)	
Use of AI tools in clinical practice, *n* (%)										
Yes	30.00 (25.00, 38.00)	**<0.001**	32.00 (27.00, 39.00)	**<0.001**	12.00 (9.00, 14.00)	**<0.001**	12.00 (11.00, 15.00)	**<0.001**	86.50 (74.25, 103.00)	**<0.001**
No	25.00 (22.00, 31.00)		28.00 (24.00, 32.00)		10.00 (9.00, 12.00)		12.00 (9.00, 15.00)		75.00 (66.00, 88.00)	

*n*: number; AI: artificial intelligence; IQR: interquartile range.

Values are presented as median (IQR). *p*-values were derived using the Mann–Whitney U test for two-group comparisons and the Kruskal–Wallis test for comparisons involving three or more groups. For variables with more than two categories, *p*-values represent omnibus tests and do not indicate specific pairwise differences. Where the omnibus test was significant, pairwise comparisons were subsequently performed using Dunn’s test with Holm adjustment (Supplementary Table S4). The univariable analyses were exploratory, and the p values presented in this table are unadjusted; Benjamini–Hochberg FDR-adjusted *p*-values are provided in Supplementary Table S3. Bold numbers in the tables indicate statistically significant differences.

**Table 3:** Multivariable linear regression analysis of factors associated with AI literacy among Chinese gastroenterologists (*n* = 1,125).

Variables	*B*	Robust 95% CI	Robust SE	*p* value
Intercept	74.47	69.29 to 79.65	2.64	**<0.001**
Sex (Ref: Male)				
Female	−0.34	−2.31 to 1.63	1.00	0.737
Age group (Ref: ≤40 years)				
>40 years	−0.20	−3.02 to 2.62	1.44	0.890
Highest degree (Ref: Bachelor’s degree)				
Master’s degree	−5.01	−7.51 to −2.52	1.27	**<0.001**
Doctoral degree	−4.11	−7.25 to −0.96	1.60	**0.011**
Professional title (Ref: Chief Physician)				
Associate Chief Physician	−1.47	−4.55 to 1.61	1.57	0.349
Attending Physician	−0.41	−4.03 to 3.22	1.85	0.825
Physician	2.40	−1.54 to 6.34	2.01	0.233
Region (Ref: Eastern Region)				
Central Region	5.41	3.05 to 7.77	1.20	**<0.001**
Western Region	−2.82	−5.29 to −0.35	1.26	**0.025**
Northeastern Region	0.02	−4.30 to 4.34	2.20	0.992
Frequency of AI tool use (Ref: rarely or never)				
2–3 times per week	3.47	0.55 to 6.38	1.49	**0.020**
4–5 times per week	5.86	2.18 to 9.54	1.88	**0.002**
Almost every day	8.00	4.71 to 11.29	1.68	**<0.001**
Received AI-related training (Ref: No)				
Yes	7.61	5.56 to 9.65	1.04	**<0.001**
Use of AI tools in clinical practice (Ref: No)				
Yes	5.93	3.73 to 8.13	1.12	**<0.001**

Model statistics: *n* = 1,125; 8 predictor variables represented by 15 regression parameters excluding the intercept; F = 22.80, *p* < 0.001; *R*^2^ = 0.236; adjusted *R*^2^ = 0.225. The model included sex, age group, highest educational attainment, professional title, geographic region, frequency of AI tool use, receipt of AI-related training, and use of AI tools in clinical practice. AI tool use frequency was modeled as a categorical variable, with “rarely or never” as the reference category. HC3 heteroskedasticity-robust standard errors were used to calculate the 95% CIs and *p*-values. Bold numbers in the tables indicate statistically significant differences.

B, unstandardized regression coefficient; SE, standard error; CI, confidence interval; AI, artificial intelligence.

Internal consistency of the Chinese MAIRS-MS was assessed using Cronbach’s *α* and McDonald’s ω for the overall scale and each domain. The original four-factor structure was examined using confirmatory factor analysis with the weighted least squares mean- and variance-adjusted (WLSMV) estimator, treating the 22 Likert-scale items as ordered categorical variables. Model fit was evaluated using the comparative fit index (CFI), Tucker-Lewis index (TLI), root mean square error of approximation (RMSEA), and standardized root mean square residual (SRMR). All tests were two-sided, with *p* < 0.05 considered statistically significant.

## 3 Results

### 3.1 Participant characteristics

A total of 1,440 questionnaires were distributed, of which 1,247 were returned, yielding a response rate of 86.60%. After 122 questionnaires were excluded, 1,125 were included in the final analysis. Of the excluded questionnaires, 56 failed the quality-control item, five were duplicate submissions, 26 exhibited invariant response patterns, and 35 were completed in less than 1 min (Supplementary Figure S1).

The mean age of the participants was 39.4 years (SD, 8.51), and 440 (39.11%) were male. Overall, 53.16% held a master’s degree, and 59.38% had more than 10 years of clinical experience. Physicians, Attending Physicians, Associate Chief Physicians, and Chief Physicians accounted for 23.38%, 31.73%, 26.76%, and 18.13% of the sample, respectively. Participants were recruited predominantly from eastern (37.33%), central (33.69%), and western China (23.73%). In addition, 37.96% had received AI-related training, and 63.64% reported using AI tools in clinical practice. The participant characteristics are summarized in Table 1.

### 3.2 AI literacy and psychometric performance of the Chinese MAIRS-MS

The median overall AI literacy score was 82 (IQR, 69–96; possible range, 22–110), corresponding to a median normalized score of 68.2% (IQR, 53.4–84.1%). The median domain scores were 28 (IQR, 24–34; possible range, 8–40) for Cognition, 31 (IQR, 26–36; possible range, 8–40) for Ability, 11 (IQR, 9–13; possible range, 3–15) for Vision, and 12 (IQR, 10–15; possible range, 3–15) for Ethics. After normalization to a 0–100 scale, the corresponding median scores were 62.5% (IQR, 50.0–81.2%), 71.9% (IQR, 56.2–87.5%), 66.7% (IQR, 50.0–83.3%), and 75.0% (IQR, 58.3–100.0%), respectively.

The Chinese MAIRS-MS demonstrated high internal consistency in the study population. Cronbach’s *α* and McDonald’s ω were 0.974 and 0.984, respectively, for the overall scale. Cronbach’s *α* values for the Cognition, Ability, Vision, and Ethics domains were 0.954, 0.953, 0.912, and 0.940, respectively, with corresponding McDonald’s ω values of 0.954, 0.954, 0.916, and 0.943.

In the CFA, standardized factor loadings for the 22 items ranged from 0.702 to 0.977 (Supplementary Table S2). The original four-factor model yielded a scaled chi-square statistic of *χ*^2^(203) = 5314.905 (*p* < 0.001), with a robust CFI of 0.859, robust TLI of 0.840, robust RMSEA of 0.157 (90% CI, 0.153–0.162), and SRMR of 0.054. Latent factor correlations ranged from 0.641 to 0.899, with the strongest correlation observed between Cognition and Ability (*r* = 0.899). Although the standardized factor loadings and SRMR were favorable, the robust CFI, TLI, and RMSEA indicated inadequate overall model fit. Thus, the CFA did not provide sufficient evidence to fully confirm the original four-factor structure in this population.

### 3.3 Factors associated with AI literacy

The results of the univariable analyses are presented in Table 2. Overall AI literacy scores differed significantly according to educational attainment, professional title, geographic region, frequency of AI tool use, receipt of AI-related training, and AI use in clinical practice (all *p* < 0.001). No significant differences were observed according to sex, age, or years in practice. Similar patterns were observed across the Cognition, Ability, Vision, and Ethics domains. Scores in all four domains differed significantly according to educational attainment, professional title, geographic region, frequency of AI tool use, AI-related training, and clinical AI use (all *p* < 0.001). In the Ethics domain, female physicians also had higher scores than male physicians (*p* = 0.034). The pattern of statistical significance was unchanged after Benjamini-Hochberg FDR correction, with all 31 comparisons that were significant based on the unadjusted *p* values remaining significant after adjustment (Supplementary Table S3). For variables with three or more categories, the Kruskal–Wallis test was interpreted as an omnibus test. When the omnibus test was statistically significant, pairwise comparisons were subsequently performed using Dunn’s test with Holm adjustment for multiple comparisons. Effect-size estimates with 95% confidence intervals for all univariable comparisons are provided in Supplementary Table S3.

The multiple linear regression model included all 1,125 participants and eight conceptually relevant predictor variables, represented by 15 regression parameters excluding the intercept. The overall model was statistically significant (*F* = 22.80, *p* < 0.001), with an *R*^2^ of 0.236 and an adjusted *R*^2^ of 0.225. Because model diagnostics indicated modest heteroskedasticity, HC3 heteroskedasticity-robust standard errors, 95% CIs, and *p* values were used for coefficient inference.

Compared with participants who rarely or never used AI tools, those who used AI tools 2–3 times per week (B = 3.47, 95% CI, 0.55 to 6.38; *p* = 0.020), 4–5 times per week (B = 5.86, 95% CI, 2.18 to 9.54; *p* = 0.002), and almost every day (B = 8.00, 95% CI, 4.71 to 11.29; *p* < 0.001) had higher AI literacy scores. Receipt of AI-related training (B = 7.61, 95% CI, 5.56 to 9.65; *p* < 0.001) and use of AI tools in clinical practice (B = 5.93, 95% CI, 3.73 to 8.13; *p* < 0.001) were also positively associated with AI literacy. Compared with participants with a bachelor’s degree, those with a master’s degree (B = −5.01, 95% CI, −7.51 to −2.52; *p* < 0.001) or doctoral degree (B = −4.11, 95% CI, −7.25 to −0.96; *p* = 0.011) had lower adjusted AI literacy scores. Compared with participants from the Eastern region, those from the Central region had higher scores (B = 5.41, 95% CI, 3.05 to 7.77; *p* < 0.001), whereas those from the Western region had lower scores (B = −2.82, 95% CI, −5.29 to −0.35; *p* = 0.025). Sex, age group, professional title, and residence in the Northeastern region were not significantly associated with AI literacy (Table 3 and Figure 1).

**Figure 1:**
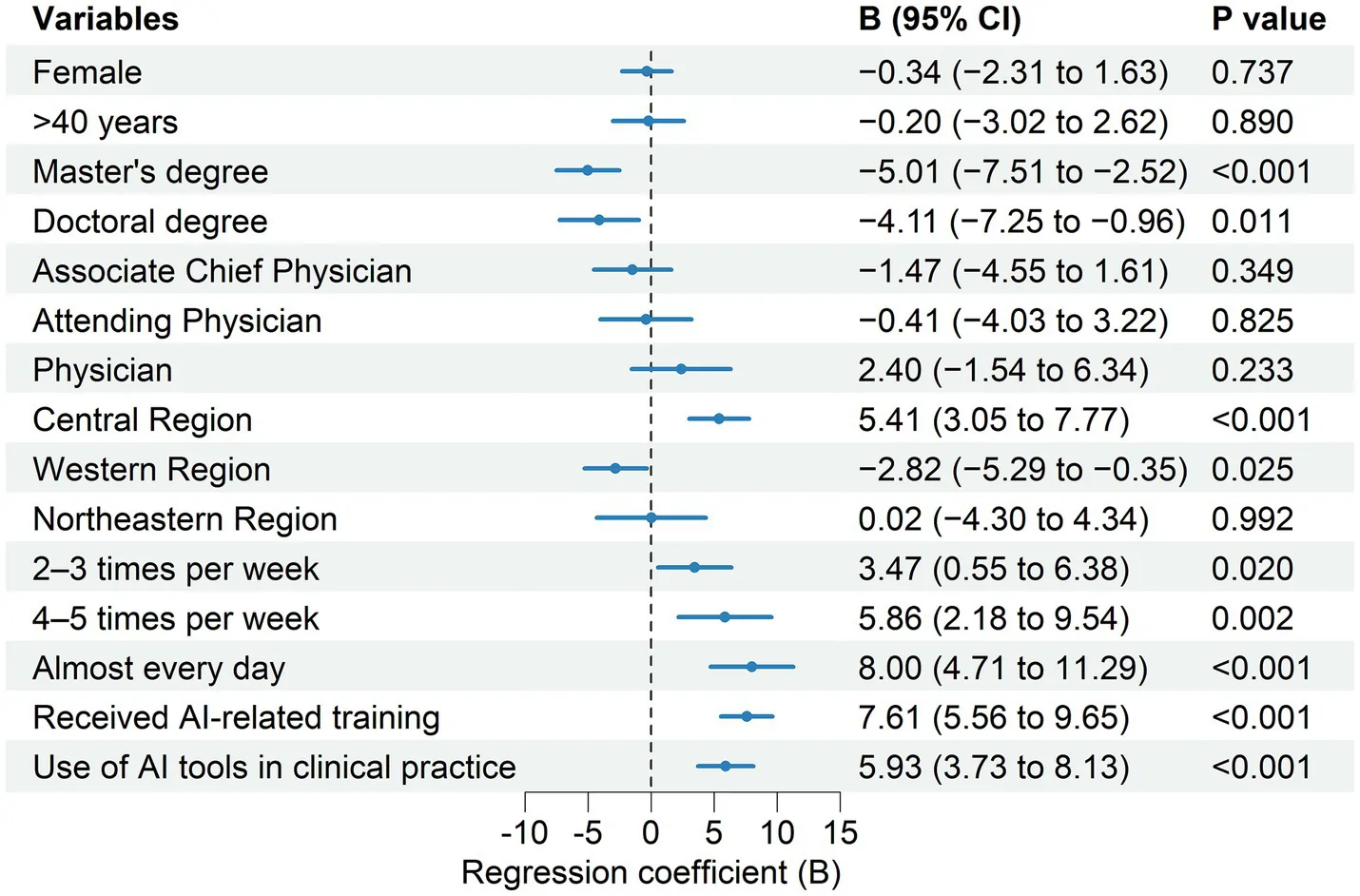
Factors associated with artificial intelligence literacy among gastroenterologists in China. Dots represent unstandardized regression coefficients (B), and horizontal lines represent HC3 heteroskedasticity-robust 95% confidence intervals. The vertical dashed line indicates a regression coefficient of zero.

Model diagnostics indicated no substantial multicollinearity, with adjusted GVIF values ranging from 1.029 to 1.445. Residuals did not significantly deviate from normality (Shapiro–Wilk W = 0.998, *p* = 0.160). The Breusch–Pagan test indicated modest heteroskedasticity (BP = 25.623, *df* = 15, *p* = 0.042). The maximum Cook’s distance was 0.018, and no observation had a Cook’s distance >1.

In the sensitivity analysis, treating AI tool use frequency as an ordinal variable showed a positive trend across increasing frequency categories (B = 2.53 per category increase, HC3 robust 95% CI, 1.56 to 3.49; *p* < 0.001). The categorical specification did not significantly improve model fit compared with the ordinal trend specification (*F* = 0.276, *p* = 0.759), indicating no evidence of substantial departure from a linear trend.

AI tool use frequency, AI-related training, and clinical AI use were significantly associated with one another (all *p* < 0.001), with Cramér’s V values ranging from 0.192 to 0.324. These associations did not result in substantial multicollinearity in the multivariable model.

## 4 Discussion

In this multicenter cross-sectional survey, the median overall MAIRS-MS score among Chinese gastroenterologists was 82 (possible range, 22–110), corresponding to a median normalized score of 68.2%. Across the four domains, the median normalized score was lowest for Cognition (62.5%), followed by Vision (66.7%), Ability (71.9%), and Ethics (75.0%). These descriptive differences suggest relatively lower self-reported readiness in the Cognition and Vision domains, although the normalized scores should not be interpreted as validated thresholds for low, moderate, or high AI literacy.

In the univariable analyses, AI literacy varied according to educational attainment, professional title, geographic region, AI tool use frequency, AI-related training, and clinical AI use. In the multivariable analysis, educational attainment, geographic region, AI tool use frequency, AI-related training, and clinical AI use remained associated with AI literacy after mutual adjustment, whereas professional title did not. Notably, AI tool use frequency, AI-related training, and clinical AI use were themselves associated with one another, suggesting that they represent related dimensions of AI exposure and engagement rather than entirely independent experiences. Their associations with AI literacy should therefore be interpreted jointly and without implying causal independence. Although these associations highlight the potential relevance of AI exposure and structured learning to physicians’ AI literacy, the cross-sectional design does not establish their temporal direction or indicate that training or more frequent AI use improves AI literacy.

Previous studies in China have focused primarily on medical students, nurses (12, 13), and nursing students (14, 15), with comparatively little attention given to practicing physicians. Surveys of Chinese nurses have generally reported moderate levels of AI literacy, broadly consistent with the present findings (13). Chinese gastroenterologists in this study obtained higher MAIRS-MS scores than participants in studies from other countries (16). However, these differences should not be interpreted as evidence of greater AI literacy among Chinese physicians.

Direct comparisons are limited by substantial differences in survey period, participant population, clinical specialty, recruitment strategy, and the language version of the instrument. Comparability may be further constrained by the rapid evolution and increasing availability of AI technologies. Studies conducted in different years may therefore capture markedly different levels of exposure, training, and practical experience. Future research should apply harmonized measurement and sampling methods across larger, more diverse populations. Such studies would enable more robust cross-national and cross-specialty comparisons and clarify how AI literacy varies across geographic regions and healthcare settings.

Notably, participants reported the lowest normalized scores in the Cognition and Vision domains, suggesting relatively lower perceived readiness in these areas. One possible explanation is the limited availability of formal AI education. Although 64% of participants reported using AI tools in clinical practice, only 38% had received AI-related training. This mismatch may suggest that exposure to AI has often occurred through task-oriented use rather than structured education, although the specific nature and quality of such AI use were not assessed in this study. Importantly, these self-reported domain scores should not be interpreted as direct evidence of deficiencies in objective AI knowledge or clinical competence. The MAIRS-MS did not objectively assess participants’ ability to understand algorithmic principles, identify model bias, verify AI-generated outputs, or use AI safely in clinical decision-making. Future studies incorporating performance-based assessments are needed to determine how self-reported AI literacy relates to these objective competencies.

More frequent AI tool use, receipt of AI-related training, and self-reported AI use in clinical practice were each associated with higher AI literacy after adjustment for the other measured covariates. For AI tool use frequency, the adjusted score differences increased progressively across the frequency categories, and the sensitivity analysis also showed a positive trend with increasing frequency. However, frequency alone provides only a limited measure of meaningful AI engagement, because more frequent use does not necessarily indicate more competent, critical, or appropriate use. Furthermore, the observed associations among AI tool use frequency, AI-related training, and clinical AI use suggest that these exposures are interrelated. Physicians who receive AI-related training may use AI more frequently or be more likely to incorporate it into their professional activities, while physicians who already engage more actively with AI may be more likely to seek additional training. The cross-sectional design does not permit these temporal pathways, including possible mediation or reverse causation, to be distinguished.

The association with clinical AI use also requires cautious interpretation. In this study, clinical AI use was assessed using a broad self-reported yes/no item and did not distinguish direct patient-care or clinical decision-support applications from other professional uses of AI. Consequently, this finding should not be interpreted as evidence that use of AI specifically for clinical decision-making is associated with greater AI literacy. Future studies should characterize the purposes, contexts, and intensity of AI use in greater detail and examine prospectively how structured training and different forms of AI engagement relate to the development of AI literacy.

Sex, age, and professional title were not independently associated with AI literacy in the multivariable analysis. Clinical experience alone may not confer competence in AI, particularly because relevant technologies and knowledge evolve rapidly. Familiarity with emerging technologies among younger physicians could potentially offset the advantages of greater clinical experience among older physicians. However, this explanation was not directly examined in our study and requires further investigation. From an educational perspective, regularly updated AI education across career stages may be worth considering, although its effectiveness in improving AI literacy requires evaluation in longitudinal or interventional studies.

After adjustment for the other measured covariates, gastroenterologists with master’s or doctoral degrees had lower AI literacy scores than those with bachelor’s degrees. This unexpected finding should not be interpreted as evidence that postgraduate education reduces AI literacy. One possible, although speculative, explanation is that respondents with postgraduate training may have assessed their competence more critically because the MAIRS-MS is a self-report instrument. Residual confounding is also possible; educational groups may have differed in professional responsibilities, prior AI exposure, institutional characteristics, or other factors not fully captured by the model. Studies using more representative samples, objective assessments, and longitudinal designs are needed to determine whether this association is reproducible and to clarify its underlying mechanisms.

Geographic variation in AI literacy was also observed after covariate adjustment. Gastroenterologists in western China had lower scores than those in eastern China, whereas those in central China had higher scores. Differences in access to AI-related infrastructure, educational resources, and continuing professional development opportunities may partly contribute to this variation; however, these potential mechanisms were not directly measured in the present study. Moreover, the regional associations should be interpreted cautiously because individual respondents could not be linked to their recruiting hospitals and hospital-level clustering could not be accounted for. The observed differences may therefore partly reflect the composition and institutional characteristics of the participating hospitals rather than geographic effects per se. Future multicenter studies incorporating hospital identifiers and institution-level indicators of AI infrastructure and educational resources are needed to disentangle geographic variation from institutional differences. If regional disparities are confirmed in such studies, national continuing professional development platforms, including standardized online courses, remote case-based discussions, and practice-oriented guidance, may provide a scalable approach to improving access to AI education in resource-limited settings.

This study extends the limited evidence on physicians’ AI literacy by providing large-sample, multicenter data from gastroenterologists across 30 provincial-level regions in China. Several limitations should be considered when interpreting the findings. First, the MAIRS-MS was originally developed for medical students rather than practicing physicians, and its measurement properties may not fully generalize to practicing gastroenterologists. Although the scale showed high internal consistency and generally strong item-level factor loadings, the robust CFA indices indicated inadequate overall fit of the original four-factor model. The very high reliability coefficients and relatively high correlations among several latent factors may also indicate some overlap among items or related constructs. Therefore, the original four-factor structure should not be considered fully validated in this population, and the domain-specific findings warrant cautious interpretation. Further psychometric evaluation in practicing physicians is needed. Second, the cross-sectional design precludes causal inference and does not establish the temporal ordering of AI literacy, AI-related training, AI tool use frequency, and clinical AI use. The observed associations may therefore reflect reciprocal relationships or reverse causation. Third, participating hospitals were not selected using probability sampling, and all were tertiary hospitals, limiting the national representativeness of the sample and the generalizability of the findings to physicians in primary- and secondary-level hospitals. In addition, although participants were nested within 96 hospitals, individual-level hospital identifiers were not collected; consequently, within-hospital clustering could not be accounted for using multilevel modeling or hospital-level cluster-robust standard errors. Shared institutional characteristics may therefore have affected the precision of some regression estimates. This limitation is particularly relevant to the regional findings, which may partly reflect differences in the composition and characteristics of the participating hospitals rather than geographic effects per se. Fourth, AI literacy was assessed using a self-report instrument and may be affected by recall, information, and social desirability biases; it should therefore not be interpreted as an objective measure of AI competence. Clinical AI use was also assessed using a single broad yes/no item that did not distinguish direct patient-care or clinical decision-support applications from other professional uses of AI. In addition, the AI tool use frequency item did not include a separate response category for approximately once-weekly use. These measurement limitations reduced the granularity with which patterns and contexts of AI use could be characterized. Finally, the exclusive inclusion of gastroenterologists limits generalizability to other medical specialties. Future studies should include physicians from more diverse specialties and healthcare settings, use probability-based sampling where feasible, collect hospital identifiers and relevant institution-level indicators, characterize specific types and contexts of AI use, and incorporate objective assessments of AI knowledge and skills alongside self-reported literacy. Longitudinal studies are also needed to clarify the temporal relationships among AI training, AI use, and AI literacy.

## 5 Conclusion

This multicenter cross-sectional survey characterized self-reported AI literacy among Chinese gastroenterologists, with the lowest normalized scores observed in the Cognition and Vision domains. Self-reported AI literacy was associated with educational attainment, geographic region, AI tool use frequency, AI-related training, and self-reported AI use in clinical practice. These observational associations highlight the potential relevance of structured, specialty-specific, and regularly updated AI education for practicing physicians, but longitudinal and interventional studies incorporating objective competency assessments are needed to determine whether such educational approaches improve AI literacy and translate into demonstrable AI-related knowledge and skills. Further research incorporating institutional characteristics is also needed to clarify the factors underlying the observed regional differences.

## Data Availability

The raw data supporting the conclusions of this article will be made available by the authors, without undue reservation.
