# Supplementary material for: Artificial intelligence literacy and associated factors among gastroenterologists in China: findings from a multicenter survey with nationwide geographic coverage
Source: Front Public Health. 2026 Oct 21;14:1967305. doi: 10.3389/fpubh.2026.1967305 (PMC13731217; doi:10.3389/fpubh.2026.1967305)
Supplement: Supplementary file 1 [file Data_Sheet_1.docx]

Artificial intelligence literacy and associated factors among gastroenterologists in China: findings from a multicenter survey with nationwide geographic coverage

**Supplementary Materials**

Yangyang Zhang^1^, Wenhao Zhang^1^, Canlin Wang^2^, Hewei Ma^1^, Lanshuo Hu^3^, Yiqing Mao^4^, Jian Lan^1^, Xudong Tang^1^, Yingpan Zhao^1^

^1^Institute of Digestive Diseases, Xiyuan Hospital, China Academy of Chinese Medical Sciences, Beijing, China.

^2^Guang’anmen Hospital, China Academy of Chinese Medical Sciences, Beijing, China

^3^Department of Gastroenterology, Tongde Hospital of Zhejiang Province, Hangzhou, China

^4^Taizhou Hospital of The First Affiliated Hospital of Zhejiang University School of Medicine, Taizhou, China

**[Supplementary Table 1. Summary of the Hospitals Participating in the Survey.	2](#_Toc13405)**

**[Supplementary Table 2 Standardized factor loadings of the Chinese MAIRS-MS among practicing gastroenterologists.	9](#_Toc26362)**

**[Supplementary Table 3. Effect sizes and multiple-testing-adjusted P values for exploratory univariable analyses of AI literacy.	11](#_Toc16250)**

**[Supplementary Table 4. Dunn post-hoc pairwise comparisons following significant Kruskal-Wallis tests.	16](#_Toc2273)**

**[Supplementary Questionnaire	28](#_Toc2086)**

**[Supplementary Figure 1. Flowchart of Participant Recruitment and Questionnaire Exclusion	30](#_Toc15193)**

# Supplementary Table 1. Summary of the Hospitals Participating in the Survey.

| **Number** | **Hospital** | **Province** | **Region** |
| --- | --- | --- | --- |
| 1 | Shenzhen Traditional Chinese Medicine Hospital | Guangdong | Eastern Region |
| 2 | The First Affiliated Hospital of Guangzhou University of Chinese Medicine | Guangdong | Eastern Region |
| 3 | Guangdong Second Traditional Chinese Medicine Hospital | Guangdong | Eastern Region |
| 4 | Dongguan Traditional Chinese Medicine Hospital | Guangdong | Eastern Region |
| 5 | Wuhan Hospital of Traditional Chinese and Western Medicine | Hubei | Central Region |
| 6 | Guangdong Traditional Chinese Medicine Hospital Fangcun Branch Hospital | Guangdong | Eastern Region |
| 7 | Shenzhen Bao'an District Traditional Chinese Medicine Hospital | Guangdong | Eastern Region |
| 8 | Shenzhen People's Hospital | Guangdong | Eastern Region |
| 9 | Jining Traditional Chinese Medicine Hospital | Shandong | Eastern Region |
| 10 | Shandong Traditional Chinese Medicine Hospital | Shandong | Eastern Region |
| 11 | Shandong Provincial Hospital | Shandong | Eastern Region |
| 12 | Dezhou People's Hospital | Shandong | Eastern Region |
| 13 | Qingdao Traditional Chinese Medicine Hospital | Shandong | Eastern Region |
| 14 | Digestive Diseases Hospital of Shandong First Medical University | Shandong | Eastern Region |
| 15 | Linyi Traditional Chinese Medicine Hospital | Shandong | Eastern Region |
| 16 | Affiliated Hospital of Jining Medical University | Shandong | Eastern Region |
| 17 | Jiangsu Province Hospital of Chinese Medicine | Jiangsu | Eastern Region |
| 18 | Jiangsu Province Integrated Traditional Chinese and Western Medicine Hospital | Jiangsu | Eastern Region |
| 19 | Nanjing Integrated Traditional Chinese Medicine and Western Medicine Hospital | Jiangsu | Eastern Region |
| 20 | Suzhou Traditional Chinese Medicine Hospital | Jiangsu | Eastern Region |
| 21 | Nanjing Traditional Chinese Medicine Hospital | Jiangsu | Eastern Region |
| 22 | Kunshan Traditional Chinese Medicine Hospital | Jiangsu | Eastern Region |
| 23 | The Second Affiliated Hospital of Zhejiang University of Chinese Medicine | Zhejiang | Eastern Region |
| 24 | Zhejiang Cancer Hospital | Zhejiang | Eastern Region |
| 25 | Hangzhou Traditional Chinese Medicine Hospital | Zhejiang | Eastern Region |
| 26 | Zhejiang Provincial Tongde Hospital | Zhejiang | Eastern Region |
| 27 | Huzhou Traditional Chinese Medicine Hospital | Zhejiang | Eastern Region |
| 28 | Hangzhou Third People's Hospital | Zhejiang | Eastern Region |
| 29 | Zhejiang Traditional Chinese Medicine Hospital | Zhejiang | Eastern Region |
| 30 | Dongfang Hospital, Beijing University of Chinese Medicine | Beijing | Eastern Region |
| 31 | Guang'anmen Hospital of China Academy of Chinese Medical Sciences | Beijing | Eastern Region |
| 32 | China-Japan Friendship Hospital | Beijing | Eastern Region |
| 33 | Dongzhimen Hospital, Beijing University of Chinese Medicine | Beijing | Eastern Region |
| 34 | Beijing Fangshan District Traditional Chinese Medicine Hospital | Beijing | Eastern Region |
| 35 | The First Affiliated Hospital of Henan University of Chinese Medicine | Henan | Central Region |
| 36 | Henan Province Hospital of Traditional Chinese Medicine | Henan | Central Region |
| 37 | Kaifeng Traditional Chinese Medicine Hospital | Henan | Central Region |
| 38 | Zhengzhou Traditional Chinese Medicine Hospital | Henan | Central Region |
| 39 | Zhejiang Provincial People's Hospital | Zhejiang | Eastern Region |
| 40 | The First Affiliated Hospital of Zhengzhou University | Henan | Central Region |
| 41 | The First People's Hospital of Zhengzhou | Henan | Central Region |
| 42 | Mianyang Traditional Chinese Medicine Hospital | Sichuan | Western Region |
| 43 | Hospital of Chengdu University of Traditional Chinese Medicine | Sichuan | Western Region |
| 44 | Leshan Traditional Chinese Medicine Hospital | Sichuan | Western Region |
| 45 | Chengdu Xinjin District Hospital of Traditional Chinese Medicine | Sichuan | Western Region |
| 46 | Chengdu Integrated TCM & Western Medicine Hospital | Sichuan | Western Region |
| 47 | Shanghai Hospital of Traditional Chinese Medicine | Shanghai | Eastern Region |
| 48 | Yueyang Hospital of Integrated Traditional Chinese and Western Medicine, Shanghai University of Traditional Chinese Medicine | Shanghai | Eastern Region |
| 49 | Longhua Hospital Affiliated to Shanghai University of Traditional Chinese Medicine | Shanghai | Eastern Region |
| 50 | Shuguang Hospital | Shanghai | Eastern Region |
| 51 | Shanghai Hospital of Integrated Traditional Chinese and Western Medicine | Shanghai | Eastern Region |
| 52 | The Second Affiliated Hospital of Fujian University of Traditional Chinese Medicine | Fujian | Eastern Region |
| 53 | Fujian Provincial People Hospital | Fujian | Eastern Region |
| 54 | Xiamen Hospital of Traditional Chinese Medicine | Fujian | Eastern Region |
| 55 | Fuzhou Traditional Chinese Medicine Hospital | Fujian | Eastern Region |
| 56 | Hebei Provincial Hospital of Traditional Chinese Medicine | Hebei | Eastern Region |
| 57 | Hengshui Hospital of Traditional Chinese Medicine | Hebei | Eastern Region |
| 58 | Handan Hospital of Traditional Chinese Medicine | Hebei | Eastern Region |
| 59 | Shijiazhuang Fifth Hospital | Hebei | Eastern Region |
| 60 | Shaanxi Provincial Hospital of Traditional Chinese Medicine | Shaanxi | Western Region |
| 61 | Affiliated Hospital of Shaanxi University of Chinese Medicine | Shaanxi | Western Region |
| 62 | Yan'an Traditional Chinese Medicine Hospital | Shaanxi | Western Region |
| 63 | Xi 'an Hospital of Traditional Chinese Medicine | Shaanxi | Western Region |
| 64 | The Affiliated Hospital of Jiangxi University of Traditional Chinese Medicine | Jiangxi | Central Region |
| 65 | Guangxi Hospital of Traditional Chinese Medicine | Guangxi | Western Region |
| 66 | The Second Affiliated Hospital of Hunan University of Chinese Medicine | Hunan | Central Region |
| 67 | Loudi Hospital Of Traditional Chinese Medicine | Hunan | Central Region |
| 68 | The Second Affiliated Hospital of Anhui University of Chinese Medicine | Anhui | Central Region |
| 69 | The First Affiliated Hospital of Anhui University of Chinese Medicine | Anhui | Central Region |
| 70 | Liaoning Hospital of TCM | Liaoning | Northeastern Region |
| 71 | Shengjing Hospital of China Medical University | Liaoning | Northeastern Region |
| 72 | Shenyang Traditional Chinese Medicine Hospital | Liaoning | Northeastern Region |
| 73 | Affiliated Hospital of Shanxi University of Chinese Medicine | Shanxi | Central Region |
| 74 | Shanxi Hospital of Traditional Chinese Medicine | Shanxi | Central Region |
| 75 | Shanxi Hospital of Integrated Traditional Chinese and Western Medicine | Shanxi | Central Region |
| 76 | The Second Affiliated Hospital of Chongqing Medical University | Chongqing | Western Region |
| 77 | Chongqing Traditional Chinese Medicine Hospital | Chongqing | Western Region |
| 78 | Yunnan Provincial Hospital of Traditional Chinese Medicine | Yunnan | Western Region |
| 79 | Kunming Municipal Hospital of Traditional Chinese Medicine | Yunnan | Western Region |
| 80 | Guizhou Provincial People's Hospital | Guizhou | Western Region |
| 81 | The Second Affiliated Hospital of Guizhou University of Traditional Chinese Medicine | Guizhou | Western Region |
| 82 | Gansu Provincial Hospital of Traditional Chinese Medicine | Gansu | Western Region |
| 83 | The Affiliated Hospital of Gansu University of Chinese Medicine | Gansu | Western Region |
| 84 | Inner Mongolia Hospital of Traditional Chinese Medicine | Inner Mongolia | Western Region |
| 85 | Baotou Mongolian and Chinese Medicine Hospital | Inner Mongolia | Western Region |
| 86 | Nangang Branch of Heilongjiang Academy of Chinese Medical Sciences | Heilongjiang | Northeastern Region |
| 87 | The First Affiliated Hospital of Heilongjiang University of Chinese Medicine | Heilongjiang | Northeastern Region |
| 88 | First Teaching Hospital of Tianjin University of Traditional Chinese Medicine | Tianjin | Eastern Region |
| 89 | The Second Affiliated Hospital of Tianjin University of TCM | Tianjin | Eastern Region |
| 90 | Affiliated Hospital to Changchun University of Chinese Medicine | Jilin | Northeastern Region |
| 91 | The Third Clinical Hospital Affiliated to Changchun University of Chinese Medicine | Jilin | Northeastern Region |
| 92 | Urumqi Hospital of Traditional Chinese Medicine | Xinjiang | Western Region |
| 93 | Xinjiang Uygur Autonomous Region Hospital of Traditional Chinese Medicine | Xinjiang | Western Region |
| 94 | Guangdong Provincial Hospital of Chinese Medicine. Hainan Hospital | Hainan | Eastern Region |
| 95 | Ningxia Medical University | Ningxia | Western Region |
| 96 | Qinghai Hospital of Traditional Chinese Medicine | Qinghai | Western Region |

# Supplementary Table 2. Standardized factor loadings of the Chinese MAIRS-MS among practicing gastroenterologists.

| **Factor** | **Item** | **Standardized loading** |
| --- | --- | --- |
| Cognition | Q1 | 0.835 |
| Cognition | Q2 | 0.798 |
| Cognition | Q3 | 0.908 |
| Cognition | Q4 | 0.922 |
| Cognition | Q5 | 0.937 |
| Cognition | Q6 | 0.966 |
| Cognition | Q7 | 0.957 |
| Cognition | Q8 | 0.859 |
| Ability | Q9 | 0.935 |
| Ability | Q10 | 0.962 |
| Ability | Q11 | 0.973 |
| Ability | Q12 | 0.967 |
| Ability | Q13 | 0.947 |
| Ability | Q14 | 0.702 |
| Ability | Q15 | 0.845 |
| Ability | Q16 | 0.888 |
| Vision | Q17 | 0.942 |
| Vision | Q18 | 0.967 |
| Vision | Q19 | 0.868 |
| Ethics | Q20 | 0.95 |
| Ethics | Q21 | 0.977 |
| Ethics | Q22 | 0.961 |

# Supplementary Table 3. Effect sizes and multiple-testing-adjusted P values for exploratory univariable analyses of AI literacy.

| **Characteristic** | **AI literacy outcome** | **Effect-size measure** | **Effect size** | **95% CI** | **Unadjusted P value** | **FDR-adjusted P value** |
| --- | --- | --- | --- | --- | --- | --- |
| Sex | Cognition | Rank-biserial correlation | 0.007 | -0.062 to 0.076 | 0.832 | 0.871 |
| Sex | Ability | Rank-biserial correlation | -0.016 | -0.085 to 0.053 | 0.647 | 0.747 |
| Sex | Vision | Rank-biserial correlation | 0.013 | -0.056 to 0.082 | 0.704 | 0.790 |
| Sex | Ethics | Rank-biserial correlation | -0.072 | -0.141 to -0.003 | 0.034 | 0.049 |
| Sex | Overall | Rank-biserial correlation | -0.013 | -0.082 to 0.056 | 0.720 | 0.790 |
| Age | Cognition | Rank-biserial correlation | 0.018 | -0.051 to 0.086 | 0.613 | 0.726 |
| Age | Ability | Rank-biserial correlation | 0.045 | -0.023 to 0.114 | 0.192 | 0.270 |
| Age | Vision | Rank-biserial correlation | 0.044 | -0.024 to 0.113 | 0.199 | 0.272 |
| Age | Ethics | Rank-biserial correlation | -0.001 | -0.069 to 0.068 | 0.985 | 0.985 |
| Age | Overall | Rank-biserial correlation | 0.035 | -0.034 to 0.103 | 0.325 | 0.430 |
| Highest degree | Cognition | Kruskal-Wallis eta-squared | 0.036 | 0.020 to 0.060 | <0.001 | <0.001 |
| Highest degree | Ability | Kruskal-Wallis eta-squared | 0.026 | 0.010 to 0.050 | <0.001 | <0.001 |
| Highest degree | Vision | Kruskal-Wallis eta-squared | 0.033 | 0.020 to 0.060 | <0.001 | <0.001 |
| Highest degree | Ethics | Kruskal-Wallis eta-squared | 0.015 | 0.004 to 0.030 | <0.001 | <0.001 |
| Highest degree | Overall | Kruskal-Wallis eta-squared | 0.034 | 0.020 to 0.060 | <0.001 | <0.001 |
| Professional titles | Cognition | Kruskal-Wallis eta-squared | 0.021 | 0.008 to 0.040 | <0.001 | <0.001 |
| Professional titles | Ability | Kruskal-Wallis eta-squared | 0.024 | 0.010 to 0.050 | <0.001 | <0.001 |
| Professional titles | Vision | Kruskal-Wallis eta-squared | 0.024 | 0.010 to 0.050 | <0.001 | <0.001 |
| Professional titles | Ethics | Kruskal-Wallis eta-squared | 0.014 | 0.004 to 0.030 | <0.001 | <0.001 |
| Professional titles | Overall | Kruskal-Wallis eta-squared | 0.027 | 0.010 to 0.050 | <0.001 | <0.001 |
| Region | Cognition | Kruskal-Wallis eta-squared | 0.069 | 0.040 to 0.100 | <0.001 | <0.001 |
| Region | Ability | Kruskal-Wallis eta-squared | 0.053 | 0.030 to 0.090 | <0.001 | <0.001 |
| Region | Vision | Kruskal-Wallis eta-squared | 0.051 | 0.030 to 0.080 | <0.001 | <0.001 |
| Region | Ethics | Kruskal-Wallis eta-squared | 0.043 | 0.020 to 0.070 | <0.001 | <0.001 |
| Region | Overall | Kruskal-Wallis eta-squared | 0.067 | 0.040 to 0.100 | <0.001 | <0.001 |
| Years in practice | Cognition | Kruskal-Wallis eta-squared | 0.000 | 0.000 to 0.007 | 0.746 | 0.799 |
| Years in practice | Ability | Kruskal-Wallis eta-squared | 0.000 | 0.000 to 0.008 | 0.553 | 0.692 |
| Years in practice | Vision | Kruskal-Wallis eta-squared | 0.000 | 0.000 to 0.008 | 0.546 | 0.692 |
| Years in practice | Ethics | Kruskal-Wallis eta-squared | 0.000 | 0.000 to 0.005 | 0.952 | 0.974 |
| Years in practice | Overall | Kruskal-Wallis eta-squared | 0.000 | 0.000 to 0.008 | 0.580 | 0.706 |
| Frequency of AI Tool Use | Cognition | Kruskal-Wallis eta-squared | 0.047 | 0.030 to 0.080 | <0.001 | <0.001 |
| Frequency of AI Tool Use | Ability | Kruskal-Wallis eta-squared | 0.076 | 0.050 to 0.110 | <0.001 | <0.001 |
| Frequency of AI Tool Use | Vision | Kruskal-Wallis eta-squared | 0.038 | 0.020 to 0.070 | <0.001 | <0.001 |
| Frequency of AI Tool Use | Ethics | Kruskal-Wallis eta-squared | 0.028 | 0.010 to 0.050 | <0.001 | <0.001 |
| Frequency of AI Tool Use | Overall | Kruskal-Wallis eta-squared | 0.062 | 0.040 to 0.100 | <0.001 | <0.001 |
| Received AI-related training | Cognition | Rank-biserial correlation | 0.410 | 0.350 to 0.466 | <0.001 | <0.001 |
| Received AI-related training | Ability | Rank-biserial correlation | 0.357 | 0.295 to 0.416 | <0.001 | <0.001 |
| Received AI-related training | Vision | Rank-biserial correlation | 0.331 | 0.268 to 0.392 | <0.001 | <0.001 |
| Received AI-related training | Ethics | Rank-biserial correlation | 0.213 | 0.146 to 0.279 | <0.001 | <0.001 |
| Received AI-related training | Overall | Rank-biserial correlation | 0.386 | 0.326 to 0.444 | <0.001 | <0.001 |
| Use of AI tools in clinical practice | Cognition | Rank-biserial correlation | 0.317 | 0.252 to 0.378 | <0.001 | <0.001 |
| Use of AI tools in clinical practice | Ability | Rank-biserial correlation | 0.340 | 0.277 to 0.401 | <0.001 | <0.001 |
| Use of AI tools in clinical practice | Vision | Rank-biserial correlation | 0.261 | 0.195 to 0.325 | <0.001 | <0.001 |
| Use of AI tools in clinical practice | Ethics | Rank-biserial correlation | 0.137 | 0.067 to 0.205 | <0.001 | <0.001 |
| Use of AI tools in clinical practice | Overall | Rank-biserial correlation | 0.324 | 0.260 to 0.385 | <0.001 | <0.001 |

**Abbreviations:** CI, confidence interval; FDR, false discovery rate; AI, artificial intelligence.

Effect sizes are reported as rank-biserial correlations (rrb) for two-group comparisons and Kruskal-Wallis eta-squared (η²H) for comparisons involving three or more groups. Confidence intervals for η²H were estimated using percentile bootstrap resampling with 5,000 replications. FDR-adjusted P values were calculated across the 45 exploratory univariable comparisons using the Benjamini–Hochberg procedure.

# Supplementary Table 4. Dunn post-hoc pairwise comparisons following significant Kruskal-Wallis tests.

| **Characteristic** | **AI literacy outcome** | **Group 1** | **Group 2** | **Z** | **Unadjusted P value** | **Holm-adjusted P value** |
| --- | --- | --- | --- | --- | --- | --- |
| Highest degree | Cognition | Bachelor's degree | Master's degree | -6.504 | <0.001 | <0.001 |
| Highest degree | Cognition | Bachelor's degree | Doctoral degree | -3.284 | 0.001 | 0.002 |
| Highest degree | Cognition | Master's degree | Doctoral degree | 2.173 | 0.030 | 0.030 |
| Highest degree | Ability | Bachelor's degree | Master's degree | -5.556 | <0.001 | <0.001 |
| Highest degree | Ability | Bachelor's degree | Doctoral degree | -3.095 | 0.002 | 0.004 |
| Highest degree | Ability | Master's degree | Doctoral degree | 1.529 | 0.126 | 0.126 |
| Highest degree | Vision | Bachelor's degree | Master's degree | -6.151 | <0.001 | <0.001 |
| Highest degree | Vision | Bachelor's degree | Doctoral degree | -4.09 | <0.001 | <0.001 |
| Highest degree | Vision | Master's degree | Doctoral degree | 0.947 | 0.344 | 0.344 |
| Highest degree | Ethics | Bachelor's degree | Master's degree | -4.207 | <0.001 | <0.001 |
| Highest degree | Ethics | Bachelor's degree | Doctoral degree | -3.162 | 0.002 | 0.003 |
| Highest degree | Ethics | Master's degree | Doctoral degree | 0.237 | 0.813 | 0.813 |
| Highest degree | Overall | Bachelor's degree | Master's degree | -6.301 | <0.001 | <0.001 |
| Highest degree | Overall | Bachelor's degree | Doctoral degree | -3.508 | <0.001 | <0.001 |
| Highest degree | Overall | Master's degree | Doctoral degree | 1.738 | 0.082 | 0.082 |
| Professional titles | Cognition | Chief Physician | Associate Chief Physician | -0.394 | 0.694 | 1.000 |
| Professional titles | Cognition | Chief Physician | Attending Physician | -0.391 | 0.696 | 1.000 |
| Professional titles | Cognition | Chief Physician | Physician | 3.605 | <0.001 | 0.001 |
| Professional titles | Cognition | Associate Chief Physician | Attending Physician | 0.017 | 0.986 | 1.000 |
| Professional titles | Cognition | Associate Chief Physician | Physician | 4.407 | <0.001 | <0.001 |
| Professional titles | Cognition | Attending Physician | Physician | 4.561 | <0.001 | <0.001 |
| Professional titles | Ability | Chief Physician | Associate Chief Physician | -1.1 | 0.272 | 0.815 |
| Professional titles | Ability | Chief Physician | Attending Physician | -0.696 | 0.486 | 0.972 |
| Professional titles | Ability | Chief Physician | Physician | 3.413 | <0.001 | 0.003 |
| Professional titles | Ability | Associate Chief Physician | Attending Physician | 0.493 | 0.622 | 0.972 |
| Professional titles | Ability | Associate Chief Physician | Physician | 4.954 | <0.001 | <0.001 |
| Professional titles | Ability | Attending Physician | Physician | 4.67 | <0.001 | <0.001 |
| Professional titles | Vision | Chief Physician | Associate Chief Physician | -1.417 | 0.156 | 0.469 |
| Professional titles | Vision | Chief Physician | Attending Physician | -0.896 | 0.370 | 0.740 |
| Professional titles | Vision | Chief Physician | Physician | 3.156 | 0.002 | 0.006 |
| Professional titles | Vision | Associate Chief Physician | Attending Physician | 0.637 | 0.524 | 0.740 |
| Professional titles | Vision | Associate Chief Physician | Physician | 5.011 | <0.001 | <0.001 |
| Professional titles | Vision | Attending Physician | Physician | 4.591 | <0.001 | <0.001 |
| Professional titles | Ethics | Chief Physician | Associate Chief Physician | 0.627 | 0.531 | 1.000 |
| Professional titles | Ethics | Chief Physician | Attending Physician | -0.039 | 0.969 | 1.000 |
| Professional titles | Ethics | Chief Physician | Physician | 3.404 | <0.001 | 0.003 |
| Professional titles | Ethics | Associate Chief Physician | Attending Physician | -0.77 | 0.441 | 1.000 |
| Professional titles | Ethics | Associate Chief Physician | Physician | 3.089 | 0.002 | 0.008 |
| Professional titles | Ethics | Attending Physician | Physician | 3.951 | <0.001 | <0.001 |
| Professional titles | Overall | Chief Physician | Associate Chief Physician | -0.572 | 0.567 | 1.000 |
| Professional titles | Overall | Chief Physician | Attending Physician | -0.507 | 0.612 | 1.000 |
| Professional titles | Overall | Chief Physician | Physician | 3.905 | <0.001 | <0.001 |
| Professional titles | Overall | Associate Chief Physician | Attending Physician | 0.094 | 0.925 | 1.000 |
| Professional titles | Overall | Associate Chief Physician | Physician | 4.931 | <0.001 | <0.001 |
| Professional titles | Overall | Attending Physician | Physician | 5.031 | <0.001 | <0.001 |
| Region | Cognition | Eastern Region | Central Region | 6.884 | <0.001 | <0.001 |
| Region | Cognition | Eastern Region | Western Region | -2.015 | 0.044 | 0.132 |
| Region | Cognition | Eastern Region | Northeastern Region | -0.649 | 0.516 | 1.000 |
| Region | Cognition | Central Region | Western Region | -8.079 | <0.001 | <0.001 |
| Region | Cognition | Central Region | Northeastern Region | -4.13 | <0.001 | <0.001 |
| Region | Cognition | Western Region | Northeastern Region | 0.469 | 0.639 | 1.000 |
| Region | Ability | Eastern Region | Central Region | 6.045 | <0.001 | <0.001 |
| Region | Ability | Eastern Region | Western Region | -1.65 | 0.099 | 0.297 |
| Region | Ability | Eastern Region | Northeastern Region | -1.025 | 0.305 | 0.610 |
| Region | Ability | Central Region | Western Region | -6.977 | <0.001 | <0.001 |
| Region | Ability | Central Region | Northeastern Region | -4.079 | <0.001 | <0.001 |
| Region | Ability | Western Region | Northeastern Region | -0.093 | 0.926 | 0.926 |
| Region | Vision | Eastern Region | Central Region | 6.058 | <0.001 | <0.001 |
| Region | Vision | Eastern Region | Western Region | -1.379 | 0.168 | 0.504 |
| Region | Vision | Eastern Region | Northeastern Region | -1.01 | 0.312 | 0.625 |
| Region | Vision | Central Region | Western Region | -6.723 | <0.001 | <0.001 |
| Region | Vision | Central Region | Northeastern Region | -4.071 | <0.001 | <0.001 |
| Region | Vision | Western Region | Northeastern Region | -0.226 | 0.821 | 0.821 |
| Region | Ethics | Eastern Region | Central Region | 4.571 | <0.001 | <0.001 |
| Region | Ethics | Eastern Region | Western Region | -2.929 | 0.003 | 0.014 |
| Region | Ethics | Eastern Region | Northeastern Region | -0.305 | 0.760 | 0.760 |
| Region | Ethics | Central Region | Western Region | -6.923 | <0.001 | <0.001 |
| Region | Ethics | Central Region | Northeastern Region | -2.617 | 0.009 | 0.027 |
| Region | Ethics | Western Region | Northeastern Region | 1.299 | 0.194 | 0.388 |
| Region | Overall | Eastern Region | Central Region | 6.641 | <0.001 | <0.001 |
| Region | Overall | Eastern Region | Western Region | -2.165 | 0.030 | 0.091 |
| Region | Overall | Eastern Region | Northeastern Region | -0.767 | 0.443 | 0.886 |
| Region | Overall | Central Region | Western Region | -8.01 | <0.001 | <0.001 |
| Region | Overall | Central Region | Northeastern Region | -4.124 | <0.001 | <0.001 |
| Region | Overall | Western Region | Northeastern Region | 0.437 | 0.662 | 0.886 |
| Frequency of AI Tool Use | Cognition | Rarely or never | 2–3 times per week | 3.141 | 0.002 | 0.005 |
| Frequency of AI Tool Use | Cognition | Rarely or never | 4–5 times per week | 4.253 | <0.001 | <0.001 |
| Frequency of AI Tool Use | Cognition | Rarely or never | Almost every day | 7.01 | <0.001 | <0.001 |
| Frequency of AI Tool Use | Cognition | 2–3 times per week | 4–5 times per week | 2.148 | 0.032 | 0.064 |
| Frequency of AI Tool Use | Cognition | 2–3 times per week | Almost every day | 5.289 | <0.001 | <0.001 |
| Frequency of AI Tool Use | Cognition | 4–5 times per week | Almost every day | 1.809 | 0.070 | 0.070 |
| Frequency of AI Tool Use | Ability | Rarely or never | 2–3 times per week | 4.196 | <0.001 | <0.001 |
| Frequency of AI Tool Use | Ability | Rarely or never | 4–5 times per week | 6.016 | <0.001 | <0.001 |
| Frequency of AI Tool Use | Ability | Rarely or never | Almost every day | 8.745 | <0.001 | <0.001 |
| Frequency of AI Tool Use | Ability | 2–3 times per week | 4–5 times per week | 3.262 | 0.001 | 0.002 |
| Frequency of AI Tool Use | Ability | 2–3 times per week | Almost every day | 6.272 | <0.001 | <0.001 |
| Frequency of AI Tool Use | Ability | 4–5 times per week | Almost every day | 1.481 | 0.139 | 0.139 |
| Frequency of AI Tool Use | Vision | Rarely or never | 2–3 times per week | 2.778 | 0.005 | 0.016 |
| Frequency of AI Tool Use | Vision | Rarely or never | 4–5 times per week | 3.196 | 0.001 | 0.006 |
| Frequency of AI Tool Use | Vision | Rarely or never | Almost every day | 6.432 | <0.001 | <0.001 |
| Frequency of AI Tool Use | Vision | 2–3 times per week | 4–5 times per week | 1.234 | 0.217 | 0.217 |
| Frequency of AI Tool Use | Vision | 2–3 times per week | Almost every day | 4.975 | <0.001 | <0.001 |
| Frequency of AI Tool Use | Vision | 4–5 times per week | Almost every day | 2.433 | 0.015 | 0.030 |
| Frequency of AI Tool Use | Ethics | Rarely or never | 2–3 times per week | 2.157 | 0.031 | 0.062 |
| Frequency of AI Tool Use | Ethics | Rarely or never | 4–5 times per week | 3.819 | <0.001 | <0.001 |
| Frequency of AI Tool Use | Ethics | Rarely or never | Almost every day | 5.235 | <0.001 | <0.001 |
| Frequency of AI Tool Use | Ethics | 2–3 times per week | 4–5 times per week | 2.531 | 0.011 | 0.034 |
| Frequency of AI Tool Use | Ethics | 2–3 times per week | Almost every day | 4.172 | <0.001 | <0.001 |
| Frequency of AI Tool Use | Ethics | 4–5 times per week | Almost every day | 0.648 | 0.517 | 0.517 |
| Frequency of AI Tool Use | Overall | Rarely or never | 2–3 times per week | 3.739 | <0.001 | <0.001 |
| Frequency of AI Tool Use | Overall | Rarely or never | 4–5 times per week | 5.115 | <0.001 | <0.001 |
| Frequency of AI Tool Use | Overall | Rarely or never | Almost every day | 8.002 | <0.001 | <0.001 |
| Frequency of AI Tool Use | Overall | 2–3 times per week | 4–5 times per week | 2.618 | 0.009 | 0.018 |
| Frequency of AI Tool Use | Overall | 2–3 times per week | Almost every day | 5.858 | <0.001 | <0.001 |
| Frequency of AI Tool Use | Overall | 4–5 times per week | Almost every day | 1.781 | 0.075 | 0.075 |

Pairwise comparisons were performed only when the corresponding omnibus Kruskal-Wallis test was statistically significant. P values were adjusted for multiple pairwise comparisons within each analysis using the Holm method. Adjusted P values <0.05 were considered statistically significant.

# Supplementary Questionnaire

**Part I. Demographic, Professional, and AI-Related Characteristics**

1. Sex (single choice)
□ Male
□ Female

2. Age: _____ years

3. Highest educational attainment (single choice)
□ Bachelor’s degree
□ Master’s degree
□ Doctoral degree

4. Professional title (single choice)
□ Chief Physician
□ Associate Chief Physician
□ Attending Physician
□ Physician

5. Province where you currently practice:

6. Years in practice (single choice)
□ <5 years
□ 5–10 years
□ >10 years

7. Frequency of AI tool use (single choice)
□ Rarely or never
□ 2–3 times per week
□ 4–5 times per week
□ Almost every day

8. Have you ever used artificial intelligence in medical practice? (single choice)
□ Yes
□ No

9. Have you ever received AI-related training? (single choice)
□ Yes
□ No

**Part II. the Medical Artificial Intelligence Readiness Scale for Medical Students (MAIRS-MS)**

The items of the MAIRS-MS are not reproduced here because the instrument is subject to copyright restrictions. The validated Chinese version was used with permission from the corresponding author of the Chinese validation study.

# Supplementary Figure 1. Flowchart of Participant Recruitment and Questionnaire Exclusion
